# Conservative Surgery in Compound Fracture of Femur (Gun-Shot Wounds)

**Published:** 1864-06

**Authors:** M. Legouest

**Affiliations:** Chirurgie Militaire, 1863


### CHRONICLE OF MEDICAL SCIENCE.

### ART. I.—*Conservative Surgery in Compound Fracture of Femur (Gun-Shot Wounds).* By M. LEGOUEST. (*Chirurgie Militaire,* 1863)

All fractures of the thigh, resulting from gun-shot wounds, have, for a long time, been considered, by the greater number of surgeons, as imperatively demanding amputation of the limb. Ribes* laid this down as an absolute rule, and his opinion, strengthened by that of Ravaton, Percy, Larrey, Dupuytren, Béguin Baudens, etc., acquired the force of law in military surgery, even while some of the illustrious surgeons just mentioned had happily departed from the principle established by themselves. Nevertheless, Fournier-Pescay,† contemporary of Ribes, wrote in 1813: "During the present epoch of military surgery, fractures of the middle of the thigh caused by fire-arms have been cured. J. L. Petit had never seen a case cured; amputation being always performed under such circumstances. The author of this article has cured five, three of which occurred at the military hospital of Brussels, in 1794. He had previously to that time, attended General Schinner who was perfectly cured." Ribes himself, returning to the Invalides after the campaigns of Russia and Saxony, was greatly astonished to see arrive at the hospital, from 1814 to 1822 seven soldiers who had had fracture of the middle of the thigh, and who had gotten well without amputation. Isolated facts, observed in campaigning—others, more numerous, gathered during our civil discords—had already shaken the conviction of surgeons, when Malgaigne boldly declared before the Academy of Medecine‡ that he repudiated, for his part, the doctrine of amputation, and that, adding his own experience to that of others, he had concluded to try and save the limbs.

The contradictory assertions touching this subject demand new investigations; since when Hutin § one of the successors of Ribes at the Hôtel des Invalides, resumed the investigations to which this surgeon had devoted himself; and the author of this book|| has ascertained what had been the relative cures of fractures of the thigh treated, on the one hand, by amputation, and on the other, by conservation of the limb, during the campaign of the East.

Hutin found at the Hôtel des Invalides, from 1847 to 1853, a large number of the old soldiers who had comminuted fractures from gun-shot wounds and who had not been amputated:

| | |
|---|---|
| Had had the femur fractured— | |
| In the lower fifth, | 10 |
| In the lower third, | 8 |
| In the middle third, below the centre, | 1 |
| At the centre, | 20 |
| In the middle third, above the centre, | 7 |
| In the upper third, | 7 |
| In the upper fourth, | 6 |
| Through the neck or the trochanters, | 4 |
| Total, | 63 |
| Giving— | |
| Fractures below the middle third, | 18 |
| Fractures within the middle third, | 28 |
| Fractures above the middle third, | 17 |
| Total, | 63 |

* Gazette Médicale de Paris, 1831, p. 101.

† Dictionaire des Sciences Médicales, art. Chirurgie Militaire, p. 101.

‡ Des Plaies d'Armes à feu, séance du 8 Mars, 1848: Paris, 1849.

§ Mémoires de Médecine, de Chirurgie et de Pharmacie Militaires, 1854.

|| Archives Générales de Médecine, t. xiii., 5e série, 1859.

Or in other words—

| | |
|---|---|
| Fractures in the centre of the femur, | 20 |
| Fractures below the centre, | 19 |
| Fractures above the centre, | 24 |
| Total, | 63 |

The fractures situated at the centre and below the centre of the femur constitute a little less than a third each of the total number of fractures of the thigh; and the fractures situated above the centre, a little more than a third of this number.

At the same time, there were 21 invalides with amputations of the thigh—

| | |
|---|---|
| For comminuted fracture of the lower fifths of the femur, | 10 |
| For comminuted fracture of the lower third, | 6 |
| For comminuted fracture of the centre, | 5 |
| Total, | 21 |

There was then no case of amputation of the thigh for fracture by fire-arms above the centre of the femur. This peculiarity, if not accidental, which it is difficult to admit, is probably due to the fact that the amputations performed high up on the thigh were followed by death. These figures show, that out of 84 fractures of the thigh from gun-shot wounds existing at the Invalides, from 1847 to 1853—63 (that is to say, three-fourths) had been treated without amputation, and 21, or the fourth, by amputation; but this approximation is not comparative, and does not give the proportion in which cases of fracture of the thigh followed by amputation or not were cured.

Our investigations concerning amputations of the thigh, consequent upon gun-shot wounds, have taught us that, during the campaign of the East, 1,664 soldiers* were amputated in the thigh

* These investigations are based upon the still unedited documents which Doctor Chenu has kindly placed at our disposition, and which were borrowed from the archives of the war office.

The number of 1,664 amputations of the thigh and of 123 cures have been established by M. Chenu in his work, which dates from the year 1859. Since this time, M. Chenu has made other investigations and gleaned new documents, which carry the number of amputations of the thigh to 1,678; those dying from the operation to 1,544, and those pensioned to 134.

Those cured were amputated—

| | |
|---|---|
| In the upper third, | 27 |
| In the middle third, | 25 |
| In the lower third, | 42 |
| Point not indicated, | 40 |
| Total, | 134 |

The number of fractures of the thigh has not been modified by the additional labors of M. Chenu; it remains at 337; that of the cures at 117, and that of the deaths at 220. The height of the seat of fractures, which was not indicated at first, is noted in the following manner:

| | Comminuted. | | Not Indicated. | | General Total. |
|---|---|---|---|---|---|
| | Pensions. | Deaths. | Pensions. | Deaths. | |
| Neck of the femur, | – | 2 | 7 | 8 | 17 |
| Trochanters, | – | 2 | 3 | 2 | 7 |
| Upper third, | 14 | 15 | 4 | 15 | 48 |
| Middle third, | 5 | 4 | 17 | 17 | 43 |
| Lower third, | 14 | 6 | 16 | 10 | 46 |
| Point not indicated, | 6 | 21 | 31 | 118 | 176 |
| | 39 | 50 | 78 | 170 | |
| | 89 | | 248 | | |
| | 337 | | | | |

These new figures only make a trifling variation in the general results of the work, which we have presented upon this subject, in the Archives Générales de Médecine, t. xiii., 5e série, p. 205.

for different lesions of the lower extremity, including therein the fractures of the femur, while 337 soldiers, having fracture of the thigh, had been treated upon the conservative plan.

| | | |
|---|---|---|
| Of the 1,664 amputated, | 123 were cured, | |
| " " " | | 1,541 died. |
| Total, . . | | 1,664 |

The 337 cases of fracture of the thigh, treated without amputation, give—

| | |
|---|---|
| Cures, . . . . . . | 117 |
| Deaths, . . . . . . | 220 |
| Total, . . . | 337 |

There is one striking fact in the results of amputation of the thigh, whatever may have been the cause, compared with those of fracture of the femur treated without amputation, it is that amputations of the thigh give five times fewer cures than non-amputated fractures of the thigh. It is very supposable, that if the 1,664 amputations of the thigh had all been operated upon for fractures of the femur, their chances of death or recovery would have been the same. In accepting this supposition, the result of our labors would be, that, in the army of the East, the chances of success for amputations has been—

| | |
|---|---|
| For the upper third, . . | 6 per 100 |
| For the middle third, . . | 6 per 100 |
| For the lower third, . . | 10 per 100 |

While the chances for recovery without amputation of fractures have been—

| | |
|---|---|
| For the upper third, . . | 31 5 per 100 |
| For the middle third, . . | 31.75 per 100 |
| For the lower third, . . | 42 per 100. |

We do not attribute an absolute value to these figures, but we believe, however, that they enable us to establish an approximative relation of the success of the treatment of fractures of the thigh without amputation to that of the treatment of fractures of the thigh by amputation. This relation is—

For the upper third of the femur, as 31.5 is to 6.
For the middle third of the femur, as 31.75 is to 6.
For the lower third of the femur, as 42 is to 10.

In a general sense, the proportion of the total, 177 survivors out of 337 wounded not amputated, is 35 per cent.; that of the total, 123 survivors out of 1,664 wounded amputated, is 7 4-10ths per cent. The relation of the totals is as 35 is to 7 4-10ths; in other words, in the Crimean war, the men treated for fractures of the thigh, by the conservative plan, got well in a proportion nearly five times greater than those treated, by amputation of the thigh, for traumatic lesions of every character of the lower extremity.

